# Two cases of possible transmitted HIV drug resistance to all currently available integrase inhibitors, the Netherlands, 2025

**DOI:** 10.2807/1560-7917.ES.2026.31.8.2600108

**Published:** 2026-02-26

**Authors:** Jeroen van Kampen, Jolanda Voermans, Widia Soochit, Thibault Mesplède, Thijs van de Laar, Wil van der Reijden, David Burger, Noga Shalev, Ard van Sighem, Daniela Bezemer, Suzanne Jurriaans, Thomas van der Vaart, Hanna de Jong, Marc van der Valk

**Affiliations:** 1 Department of Viroscience, Erasmus University Medical Center, Rotterdam, the Netherlands; 2 Department of Medical Microbiology and infectious Diseases, Erasmus University Medical Center, Rotterdam, the Netherlands; 3 Department of Medical Microbiology, OLVG Lab, Amsterdam, the Netherlands; 4 Laboratory of blood-borne infections, Sanquin Research, Amsterdam, the Netherlands; 5 Department of Pharmacy, Pharmacology & Toxicology, Radboud University Medical Center, Nijmegen, the Netherlands; 6 Department of infectious diseases, and Amsterdam Institute for Immunology and Infectious Diseases (AII), Amsterdam UMC location University of Amsterdam, Amsterdam, the Netherlands; 7 Stichting hiv Monitoring, Amsterdam, the Netherlands

**Keywords:** HIV, transmitted drug resistance, dolutegravir, bictegravir, cabotegravir, INSTI

## Abstract

Transmitted integrase inhibitor resistance in newly diagnosed HIV remains rare. We report two cases with baseline resistance to all currently available integrase inhibitors in the Netherlands in 2025. Clinical history and laboratory findings indicate possible transmitted resistance. The cases are not phylogenetically linked, representing two independent introductions of integrase inhibitor resistant HIV into the Dutch population. These observations highlight the need for strengthened surveillance and prevention efforts in Europe and globally.

Combination therapies containing integrase strand transfer inhibitors (INSTIs) are widely used for HIV treatment worldwide. Here we explore the possibility of transmitted INSTI resistance and aim to identify transmission clusters in two individuals newly diagnosed with HIV-1 in the Netherlands in 2025.

## Case reports

In Q3 of 2025, two people were diagnosed with HIV-1 resistant to all currently available INSTIs in the Netherlands. Case 1 concerns a heterosexual man from the Netherlands who was tested for HIV because of chronic pain. Case 1 reported not having any sexual partners since 2022. Case 2 is a bisexual man born in South America, who had emigrated to the Netherlands some months before HIV diagnosis. Both cases reported no prior HIV testing and no use of antiretroviral drugs for HIV treatment, pre-exposure prophylaxis (PrEP) or post-exposure prophylaxis (PEP).

## Laboratory analyses

We performed genotypic resistance analyses of reverse transcriptase (RT), protease, integrase and 3-polypurine tract (3’-PPT) with Sanger sequencing. For Case 1, sequencing was confirmed with Illumina Next-Generation Sequencing (Illumina, San Diego, the United States (US)). Mutations were scored from 20% to 3% of the virus population using the Stanford University HIV drug resistance database (HIVdb; https://hivdb.stanford.edu/) based on consensus sequences derived by read-mapping to an HIV vector pNL4–3 proviral HIV-1 reference sequence [1]. Sequencing results were confirmed with Sanger on a second plasma sample obtained 23 days after the first sample. Subtyping of the virus was performed with sequences of RT, protease and integrase using the COMET HIV-1 subtyping database (https://comet.lih.lu/). Plasma concentrations of antiretroviral drugs were determined using liquid chromatography–mass spectrometry (LC-MS/MS) [2,3].

In the samples of Case 1, CD4^+^ T-cell count at diagnosis was 220 cells/mm^3^ and plasma HIV-1 RNA was 17,000 copies/mL (Table). Baseline resistance testing revealed HIV-1 subtype B harbouring the Q148H + G140S integrase mutations, conferring intermediate level resistance to dolutegravir and bictegravir and high level resistance to cabotegravir, raltegravir and elvitegravir (resistance levels as defined by the Stanford HIVdb algorithm) [1,4-6]. No resistance mutations were detected in the protease or RT genes. Treatment with a protease inhibitor-containing regimen was started (darunavir/cobicistat/tenofovir alafenamide/emtricitabine) which resulted in plasma HIV-1 RNA declining to 159 copies/mL after 35 days of treatment.

**Table:** Selected characteristics of cases with drug-resistant HIV, the Netherlands, 2025 (n = 2)

Characteristic	Case 1	Case 2
CD4^+^ T-cell count (cells/mm^3^)	220	360
Prior PrEP use	No	No
HIV-1 subtype	B	B
Plasma viral load (copies/mL)		
First sample (at detection)	17,000	9,200
Second sample	32,000 (23 days)	81 (76 days)^a^
Third sample	159 (58 days)^a^	< 20 (174 days)^a^
Resistance testing		
INSTI-RAM	G140S, Q148H	E138K, Q148K
NRTI-RAM	None detected	None detected
NNRTI-RAM	None detected	K101E, Y181C, H221Y
PI-RAM	None detected	None detected
3’-PPT-RAM	None detected	Not available
Dolutegravir	< 0.01 mg/L	Not available
Bictegravir	< 0.02 mg/L	Not available
Cabotegravir	< 0.05 mg/L	< 0.05 mg/L
Raltegravir	< 0.01 mg/L	Not available
Elvitegravir	< 0.05 mg/L	Not available
Rilpivirine	< 0.003 mg/L	< 0.003 mg/L

INSTI: integrase strand transfer inhibitor; NNRTI: non-nucleoside reverse transcriptase inhibitor; NRTI: nucleoside reverse transcriptase inhibitor; PI:  protease inhibitor; PrEP: pre-exposure prophylaxis; 3’-PPT: 3’-polypurine tract; RAM: resistance-associated mutation.

^a^ Sample taken during HIV treatment, the number of days refers to days after the first sample.

HIV immunoblotting was conducted with INNO-LIA HIV I/II Score assay (Fujirebio, Tokyo, Japan). For Case 1, the samples were positive for GP120, GP41, p31, p24 and p17, and negative for GP105 and GP36; for Case 2: positive for GP160/120, GP41, p17, p24, p31, p51 and p66, negative for HIV-2 and p55.

In the samples of Case 2, CD4^+^ T-cell count at diagnosis was 360 cells/mm^3^ and plasma HIV-1 RNA was 9,200 copies/mL. Baseline resistance testing revealed HIV-1 subtype B harbouring the Q148K + E138K integrase mutations conferring intermediate level resistance to dolutegravir and bictegravir and high level resistance to cabotegravir, raltegravir and elvitegravir [1,4-6]. In the RT gene, the K101E + Y181C + H221Y mutations were detected, conferring resistance to non-nucleoside reverse transcriptase inhibitors [1,4]. No resistance mutations were detected in the protease gene. Treatment with a protease inhibitor-containing regimen (darunavir/cobicistat/tenofovir alafenamide/emtricitabine) was started which resulted in plasma HIV-1 RNA load declining to below 20 copies/mL after 174 days of treatment.

## Cluster analysis

To investigate whether Case 1 and Case 2 were part of known transmission clusters within the Netherlands, we performed a phylogenetic analysis using protease/RT sequences of these two cases and all sequences (n = 13,903) available in the national database of the Stichting HIV monitoring (SHM; https://www.hiv-monitoring.nl/en) (Figure).

**Figure:**
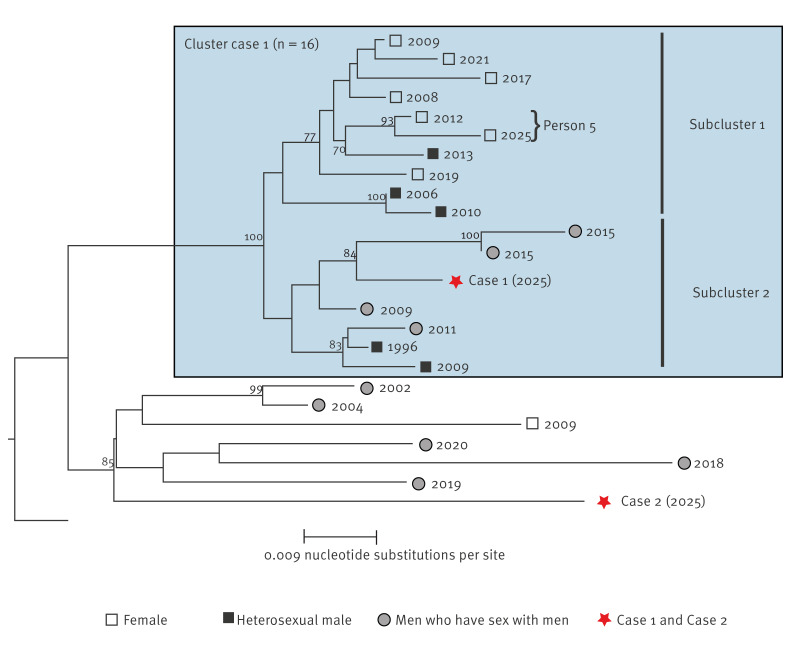
Phylogenetic cluster analysis of HIV sequences, the Netherlands, 1981–2025 (n = 13,905)^a^

RT: reverse transcriptase; SHM: Stichting HIV monitoring (https://www.hiv-monitoring.nl/en).

^a^ In the figure, sequences of Case 1, Case 2 and 22 (of 13,093) most closely related HIV sequences obtained from the SHM monitoring database are presented.

The phylogenetic tree contains one HIV sequence per person, and year of sequencing equals the year of HIV diagnosis. For Person 5, an additional sequence was obtained in 2025, and it was added to represent the HIV strain with treatment-emergent INSTI resistance (G140S + Q148H + N155H).

Clusters containing Case 1 and Case 2 were identified in FastTree analysis [17] with HIV-1 polymerase sequences trimmed at 1,200 nt from 13,903 sequences in the SHM, including at least the first 600 nt of RT. Maximum-likelihood phylogenetic trees of HIV-1 pol (polymerase) nt sequences were inferred using PhyML version 3.1 [18] implemented in SeaView version 5.1 [19], under a GTR substitution model with four discrete gamma-distributed rate categories (GTR + Γ4). Branch support was assessed using 1,000 bootstrap replicates. Bootstrap percentages > 70% are shown at the nodes.

Case 1 sequences clustered with those from 15 persons in the SHM database, of whom one person (Person 5) had treatment-emergent INSTI resistance (G140S + Q148H + N155H) due to suboptimal treatment adherence in 2025. It is highly improbable in terms of phylogenetic analysis that HIV transmission occurred between Case 1 and Person 5: the number of protease/RT and integrase nucleotide differences were 39/1,188 (3.3%) and 20/867 (2.3%), respectively. Furthermore, timelines argue against transmission between these two persons as Person 5 had treatment-emergent INSTI resistance in 2025 while Case 1 was diagnosed with a long-standing chronic HIV infection in 2025. This may indicate transmission from persons with HIV who are not yet diagnosed, or people may have opted out of the SHM database or no genotypic resistance testing was performed. Case 2 had a singleton HIV sequence, not closely related to any of the sequences in the SHM database. Taken together with his recent migration to the Netherlands and low CD4^+^ T-cell count at diagnosis, Case 2 may have acquired HIV in another country. To further explore HIV acquisition, an additional BLAST with sequences from the Los Alamos HIV Sequence Database was performed. Closest sequences had > 4% nt differences and no specific geographic region was identified in the ten most closely related sequences. Contact tracing did not provide further leads in both cases.

## Discussion

In the Netherlands, HIV drug resistance is monitored by SHM. In the most recent SHM analysis, including data obtained from all people newly diagnosed with HIV in the Netherlands between 2015 and 2024, no clinically significant transmitted drug resistance to dolutegravir or bictegravir was identified (789 baseline integrase sequences analysed) [7]. One person had HIV-1 subtype B with the G140S mutation in integrase resulting in resistance to the first generation INSTIs raltegravir and elvitegravir, but not in clinically relevant resistance to dolutegravir and bictegravir (clinically relevant resistance was defined as at least low level resistance). In addition, three persons had HIV-1 subtype B with the E138K integrase mutation, which is associated with potential low level resistance to dolutegravir and bictegravir, and low level resistance to raltegravir, elvitegravir and cabotegravir.

Various aspects of the two cases identified in 2025 are concerning. Firstly, the two cases are not phylogenetically linked and thus represent two separate introductions of HIV resistant to all currently available INSTIs into the Dutch population. Secondly, clinical history and laboratory results suggest that both cases had long-standing HIV infection with prolonged replication of drug-resistant HIV without reversion to wild type. This observation may indicate that the two viruses are relatively fit or that the identified INSTI resistance mutations are fixed in the genome due to compensatory mutations, with potential implications for their transmissibility [8,9]. While the Q148H and Q148K mutations in integrase reduce the in vitro HIV fitness, this defect is partially restored by the G140S compensatory mutation [10]. However, the in vivo fitness of the viruses from Case 1 and Case 2 cannot be inferred from epidemiological and genotypic data alone and would require further experimentation. Thirdly, clinical studies have shown that mutations at position Q148 in combination with other INSTI resistance mutations, as observed in our cases, are associated with failure to achieve virological suppression in people treated with dolutegravir-based antiretroviral therapy (ART) [5,6]. Fourthly, viruses with the Q148H + G140S or Q148K + E138K integrase mutations are also resistant to cabotegravir, which was recently approved in the Netherlands as long-acting HIV PrEP.

Both cases reported not having been diagnosed with HIV in the past and not using ART in the past. In addition, the concentrations of antiretroviral drugs tested at the time of diagnosis were below the detection limit, indicating that at that moment no ART was taken. It should be noted that these findings do not prove transmitted drug resistance. The clinical history and laboratory findings may indicate possible transmitted drug resistance in both cases. To further substantiate the possibility of transmitted drug resistance, characterisation of the proviral reservoir would be needed. However, peripheral blood mononuclear cells for proviral reservoir characterisation were not available for the two cases and is not part of routine clinical care in the Netherlands.

Treatment regimens containing the second-generation INSTIs dolutegravir or bictegravir are the most frequently used regimens in Europe. In 2018, the World Health Organization recommended replacing efavirenz-based ART with dolutegravir-based ART in low- and middle-income countries, due to concerns over the increasing prevalence of transmitted efavirenz resistance. This recommendation led to the wide-scale use of dolutegravir, with currently more than 25 million persons with HIV treated with dolutegravir-based combination therapy. To date, documented transmission of dolutegravir- and bictegravir-resistant HIV-1 has been limited [11-13]. However, modelling suggested that transmitted dolutegravir resistance may increase to 5% in 2035 in certain settings [14]. Resistance to second generation INSTIs may significantly compromise the efficacy of currently recommended ART. Our findings warrant coordinated efforts to monitor and mitigate the spread of transmitted INSTI resistance across Europe and globally [15]. These efforts should include genotypic resistance testing with sensitive technologies such as deep sequencing to estimate the true burden of HIV drug resistance [16].

## Conclusion

In Q3 of 2025, two individuals were newly diagnosed with HIV-1 resistant to all currently approved integrase inhibitors, while such resistant viruses at baseline were previously unreported in the Netherlands. Integrase inhibitor resistance may considerably compromise the efficacy of currently recommended HIV treatment regimens as well as PrEP with integrase inhibitors. These findings highlight the need for strengthened surveillance and prevention efforts in Europe and globally.

## Data Availability

The individual-level dataset analysed in this study cannot be shared publicly because of legal restrictions. Sequences are available upon reasonable request (with a data transfer agreement).
